# Mothers’ experience of their contact with their stillborn infant: An interpretative phenomenological analysis

**DOI:** 10.1186/1471-2393-14-203

**Published:** 2014-06-13

**Authors:** Kirsty Ryninks, Cara Roberts-Collins, Kirstie McKenzie-McHarg, Antje Horsch

**Affiliations:** 1 Psychological Health Service, University Hospitals Bristol NHS Foundation Trust, Level 6 Paul O’Gorman Building, Upper Maudlin Street, Bristol BS2 8BJ, UK; 2 Department of Psychology, University of Bath, Claverton Down, North East Somerset BA2 7AY, Bath, UK; 3 Department of Clinical Health Psychology, Warwick Hospital, Lakin Rd, Warwick CV34 5BW, UK; 4 Department of Child and Adolescent Psychiatry, Research Unit, University Hospital Lausanne, Rue du Bugnon 25 A, Lausanne CH-1011 Lausanne, Switzerland; 5 Department of Neonatology, University Hospital Lausanne, Avenue Pierre-Decker 2, CH-1011 Lausanne, Switzerland

**Keywords:** Stillbirth, Mothers, Perinatal loss, Grief, Qualitative research, IPA, Maternal mental health

## Abstract

**Background:**

Guidelines surrounding maternal contact with the stillborn infant have been contradictory over the past thirty years. Most studies have reported that seeing and holding the stillborn baby is associated with fewer anxiety and depressive symptoms among mothers of stillborn babies than not doing so. In contrast, others studies suggest that contact with the stillborn infant can lead to poorer maternal mental health outcomes. There is a lack of research focusing on the maternal experience of this contact. The present study aimed to investigate how mothers describe their experience of spending time with their stillborn baby and how they felt retrospectively about the decision they made to see and hold their baby or not.

**Method:**

In depth interviews were conducted with twenty-one mothers three months after stillbirth. All mothers had decided to see and the majority to hold their baby. Qualitative analysis of the interview data was performed using Interpretive Phenomenological Analysis.

**Results:**

Six superordinate themes were identified: Characteristics of Contact, Physicality; Emotional Experience; Surreal Experience; Finality; and Decision. Having contact with their stillborn infant provided mothers with time to process what had happened, to build memories, and to ‘say goodbye’, often sharing the experience with partners and other family members. The majority of mothers felt satisfied with their decision to spend time with their stillborn baby. Several mothers talked about their fear of seeing a damaged or dead body. Some mothers experienced strong disbelief and dissociation during the contact.

**Conclusions:**

Results indicate that preparation before contact with the baby, professional support during the contact, and professional follow-up are crucial in order to prevent the development of maternal mental health problems. Fears of seeing a damaged or dead body should be sensitively explored and ways of coping discussed. Even in cases where mothers experienced intense distress during the contact with their stillborn baby, they still described that having had this contact was important and that they had taken the right decision. This indicates a need for giving parents an informed choice by engaging in discussions about the possible benefits and risks of seeing their stillborn baby.

## Background

Stillbirth, defined in the United Kingdom (UK) as intra-uterine death after 24 weeks gestation, is a devastating experience for women, their partners, and the extended family. In addition to the grief that a mother and her partner experience following the discovery that their baby has died in utero or during labour, stillbirth has also been found to trigger feelings of worthlessness, isolation and guilt [1-3]. Psychiatric symptoms are also common, including depression, anxiety, posttraumatic stress and traumatic grief [4-8]. Additionally, parents report adverse long-term effects on their ability to manage their jobs and their family life [9].

Research focusing on the clinical management of mothers in the UK who have experienced a stillbirth has been contradictory for several decades. Prior to the 1970s, management of stillbirths involved removing the stillborn baby in an attempt to minimise psychological trauma and avoid parental distress at the sight of the dead body [10]. Although the best interests of the parents were at heart, parents reported that health professionals did not acknowledge the severity of their loss and that they felt they had no permission to talk about the death. Lewis [11] first criticised this approach and many researchers reported narratives of mothers regretting not having contact with their babies and suffering long term problems following suppression of their grief, as well as a prolonged mourning process [12-16].

Following anecdotal evidence from grieving mothers and health professionals, the Royal College of Obstetricians and Gynaecologists in the UK published guidelines encouraging parents to see and hold their stillborn baby, hold a funeral and to keep mementoes [17]. In the years that followed, a number of studies supported this recommendation, reporting that seeing and holding a stillborn baby resulted in fewer anxiety and depressive symptoms and improved psychological wellbeing in mothers of stillborn babies than those who did not have such contact [18-21]. In addition, Surkan and colleagues [22] reported that mothers who were with their stillborn baby for as long as they desired had lesser depressive symptoms when compared to mothers who were not with their baby for as long as they wished.

In contrast, studies by Hughes and colleagues [23,24] suggested that rather than aiding healthy grieving and psychological adjustment, behaviours promoting contact with the stillborn infant were associated with poorer outcomes. Mothers who held their stillborn infant were significantly more depressed than those who only saw the infant, while those who did not see the infant were least likely to be depressed (39% vs. 21%). However, these studies defined stillbirth as occurring at 18 weeks gestational age or later and the impact of seeing an earlier fetus is likely to be different from seeing a stillborn baby at or near term.

Current UK guidelines state that 'mothers whose infants are stillborn or die soon after birth should not be routinely encouraged to see and hold the dead infant' [25]. Although for many mothers, stillbirth leads to intense distress, it remains unclear whether seeing a baby after a stillbirth is a positive or negative experience for them. Whilst the majority of published articles report findings from quantitative studies, few studies have used qualitative methods to investigate maternal contact with stillborn babies. Those that have used qualitative methods have examined emotional responses to perinatal loss [26], interactions with staff [27], and fetal abnormality [28]. The current study aimed to develop a better understanding of mothers’ experiences of spending time with their stillborn baby, the psychological impact of seeing and holding the baby and how they feel about the decision they made at the time to see their baby or not.

## Methods

### Theoretical basis

A qualitative methodology was employed as it provides participants with scope to discuss personal experiences in depth. It can also achieve a specific and deep knowledge of an issue and has the potential to capture concepts that may not have previously been identified [29-33]. Interpretive Phenomenological Analysis (IPA) was used as it enables the researcher to explore, flexibly and in detail, an area of concern [31]. IPA is concerned with an individual’s subjective report, rather than formulating an objective account and is therefore considered phenomenological; furthermore, research is recognised as a dynamic process [34]. Within this dynamic process the researcher plays an active role by taking an ‘insider’s perspective’ [35] to explore the essence of the participant’s experience and engage in new areas of knowledge. The researcher also uses their own conceptions to interpret this information [31]. This method of the participant interpreting their own life and the researcher interpreting this knowledge is known as a “double hermeneutic” [36]. Although the interpretation of the researcher could be seen as a disadvantage of the methodology, this flexibility and ability to engage in new areas allows the researcher to become immersed within the data whilst acknowledging their own values. This is a key strength of the IPA approach [37].

IPA was considered a more suitable approach for this study than other qualitative methods because it recognises that our experience of the world is strongly influenced by personal, societal, cultural and familial perspectives [38]. IPA captures an individual’s personal experience and their world, instead of creating a “theoretically saturated” account to tie into the research question [32]. It enables a framework to be developed, classifying and organising information according to key themes, concepts and emergent categories. Consequently, interpretations are bounded by the participant’s ability to articulate their thoughts and experiences and the researcher’s ability to reflect upon and analyse the conversation [39].

### Study design and participants

Participants were recruited from nine National Health Service (NHS) hospitals in the UK and approached via their bereavement midwife who had received training, information and support from study personnel to ensure that they were comfortable explaining the purpose and protocol of the study. Women 18 and over who had experienced a stillbirth at 24 weeks gestational age or later and who were not pregnant again 3 months after stillbirth were eligible to participate. Main ethical approval was granted from the Oxfordshire research ethics committee B (study number: 06/Q/605/15) and site specific approval for eight other sites (Northampton, Swindon, Reading, High Wycombe, Bristol, Milton Keynes, Warwick, and Aylesbury).

This study formed part of a longitudinal (Stillbirth and Mental Health in Women, SAMMI) study investigating cognitive predictors and risk factors for post-traumatic stress disorder (PTSD), its time course and relationship with perinatal grief in mothers following stillbirth. This involved mothers completing structured clinical interviews and questionnaires assessing PTSD and perinatal grief symptoms, cognitive predictors, and risk factors at 3 and 6 months after stillbirth [40].

Mothers who were interested were given an information pack by bereavement midwives to take home so they could discuss the study with family and friends, and make a decision over time as to whether or not they would like to participate. No mother was approached again if she chose not to participate, and researchers remained unaware of potential participants until they themselves opted in by contacting the study office with a consent form. Hospitals chose to either hand out consent forms which immediately allowed mothers to choose to join the study, or to distribute consent forms which said that mothers would like further information, in which case these mothers were sent more details and a further consent form to join the study.

Once a consent form was received, the study administrator personally contacted the woman via her stated preferred method of contact. The administrator provided the woman with an opportunity to ask further questions, and sensitively established the date of the woman's stillbirth. This then defined the interview date; all interviews took place at 3 months post-stillbirth +/− 1 week in participants’ homes. This meant that they could feel more relaxed, safe, and open to talk in their own environment, which was particularly important due to the sensitivity of the information being discussed. The five researchers involved in undertaking the interviews were highly aware of the potential emotional challenges of the interviews and remained empathic to this at all times. Additionally, all women were sent a resource list following the interview offering further sources of support. This included information about support groups, charities, books, and websites about where they could access any extra help.

All interviews were audio recorded with the woman's consent and lasted between 20 and 30 minutes. The interviews were semi-structured and aimed to elicit information about the woman's experience of the stillbirth, as well as detailed information about her choices regarding contact with her stillborn baby. Two open-ended questions were used: ‘How would you describe your experience of spending time with your baby?’ and ‘Looking back, how do you feel about the decision you took at the time about spending time with your stillborn baby?’.

### Data collection and analysis

All transcribed interviews were anonymised and pseudonyms added in order to ensure confidentiality and privacy of participants. The interview data was analysed manually to classify and code information according to key themes, concepts and emergent categories using IPA [31].

Transcripts were repeatedly read and listened to by researchers to become as familiar and intimate as possible with each participant’s account (KR, CRC). The first transcript was read through a number of times, with initial notes of associations and possible codes, summarising the experiences that a participant described during the interview, noted in the left-hand margin. After reading the transcript again, several theme titles that emerged were noted in the right-hand margin. Key words and phrases from participants were used to label themes, maintaining the phenomenological nature of IPA. This was repeated several times using the transcript content, initial notes and themes. A preliminary list of themes which emerged from the transcript was compiled and examined to see if they could be meaningfully grouped together and clustered by shared meaning. Each interview was analysed using the same process until all transcripts had been analysed. As new clustering of themes emerged, the researchers checked back to the transcripts to ensure the connections worked for the primary source material. As the analysis progressed, themes were updated and clustered by shared meaning to develop and tabulate superordinate and master themes. The master themes maintained shared meaning across analyses when checked back against the original transcripts. Credibility checks were achieved through triangulation with senior members of the research team (KM, AH).

## Results

### Sample characteristics

Twenty-one mothers with a mean age of 34.4 years (SD = 5.2) were interviewed as part of a larger longitudinal study of 65 mothers, which investigated maternal mental health following stillbirth [40]. In the current sample, stillbirths occurred on average at 35.17 weeks gestational age (SD = 5.2). All women had seen their stillborn baby and 19 (90.5%) had held their baby. The total average number of pregnancies (including the stillbirth) was 2.0 (SD = 1.2). Before the current stillbirth, 3 (14%) of the women interviewed had had a miscarriage, 4 (19%) had had a termination and one woman (4.8%) had had a previous stillbirth. Further demographic information is given in Table 1.

**Table 1:** Sample characteristics (N = 21)

	**Mean (SD) or N (%)**
**Age** (years)	35.43 (5.22)
**Marital status**	
Married	14 (66.67 %)
Co-habiting	7 (33.33 %)
**Ethnic background**	
White UK	18 (85.17 %)
White other	2 (9.52 %)
Other	1 (4.76 %)
**Employment status**	
Full-time	12 (57.14 %)
Part-time	5 (23.81 %)
Home-maker	2 (9.52 %)
Unemployed	2 (9.52 %)
**Household income**	
£0-19,999	2 (9.52 %)
£20-29,999	2 (9.52 %)
£30-39,999	5 (23.81 %)
£40-49,999	4 (19.05 %)
£ + 50,000	8 (38.1 %)
**Total number of pregnancies** (including stillbirth)	2.0 (1.2)
**Weeks of gestation at stillbirth**	35.17 (5.21)
**History of previous pregnancy loss**	
Miscarriage	3 (14.29 %)
Stillbirth	1 (4.76 %)

Six superordinate themes and 15 subordinate themes were developed (see Table 2). These themes will be discussed in turn using direct quotations to illustrate them. Additional quotes to the ones presented in the text are given in Table 2.

**Table 2:** Superordinate and Subordinate themes

**SUPERORDINATE**	**SUBORDINATE**
1. Characteristics of Contact	1.1 Having Time
	*I guess having some time and then seeing her was quite good. You feel like you’re, you’re coming to a bit more. I think if we’d have seen her too soon after I wouldn’t have been really quite with it enough (Sophie)*
	1.2 Shared experience
	*They dressed him. (Partners) parents came over to be with us. When (partner) and I were together we really dwelled. When other people were there we chatted about other stuff. My mum and dad were in the delivery suite waiting. (Partners) mum wanted to see him, dad wasn’t sure. We didn’t want to put pressure on them, they had to do it for themselves, then it was all of us together. It was nice that all of them came and they shared that with us. It’s a shared experience (Emma)*
2. Physicality	2.1 Fear of a dead body
	*It was preparing for what was he going to look like, were we going to feel a bond with him, or were we going to feel disgust, we were worried and concerned about that (Emma)*
	2.2 Relief at well-formed body
	*It was good to see that you know he was really well formed (Charlotte)*
	2.3 Identifying family traits
	*Holding her, seeing what she looked like, knowing whether she looked like me or like (partner). This might sound strange but I wondered if she’d have a crossover toe like me but she didn’t. Her hair was like her dad’s, dark and curly. You pin all your hopes on what they’ll be like and I feel robbed of it. If I hadn’t seen her it’d be 10 times worse as I’d never have known her. I can be at peace knowing that I’d held her. I needed that (Hannah)*
	2.4 Damage
	*'Cause at one stage they kept her in a cool room, they didn’t call it that but it obviously was, so when we had her with us and she was obviously warming up a little bit, some blood was coming out of her nose, it was like she was alive in a way, you know, and every time I moved she moved and it was like she could still be there, but she obviously wasn’t (Olivia)*
3. Emotional Experience	3.1 Positive experience/process
	*And when I was young I'd always had, I was always very scared of death, I remember being somewhere and sitting on my mum’s lap and crying and crying because I didn’t want them to die and sort of a real morbid. I was so scared of death really. I just think all of that was put to rest when I had (daughter), thinking it’s not so scary really is it, just one of those things that happens, a dead body is a dead body and there is nothing to be worried about so in that way it was quite a good thing that allayed a lot of fears for me about death (Olivia)*
	3.2 Maternal Pride
	*I did, I did (feel like a proud mum)…it was a positive experience yeah, obviously I was sad, you know, you know, but the main thing I felt when I held him was, was being proud that he was that little person and that he was mine (Lucy)*
	3.3 Grief
	*It’s sad. It just feels as if there is nothing you can do (Lucy)*
4. Surreal Experience	4.1 Disbelief
	*Because all the time when we were at the hospital I didn’t react emotionally so I think when they told me, when they actually confirmed that he had died, one of my first questions was “when can I try for a baby again?” why, I don’t know why, but it didn’t register I don’t think, what had happened, and for me, all I was thinking about was actually giving birth to my baby so I, obviously I was very upset but I didn’t really think about, I don’t think I really put it into context what had happened (Lucy)*
	4.2 Dissociation
	*My experience of it? It just seems blurred like it was a dream (Victoria)*
5. Finality	5.1 Saying goodbye and Feeling sorry
	*It was just being able to say goodbye to her properly, getting memories and things to remember her by, and just having cuddles and things. It was a special time (Katherine)*
	5.2 Realising/accepting death
	*Helps you to cope with what happened (Rebecca)*
6. Decision	6.1 Satisfied with decision
	*I think I would have felt worse now if I hadn’t, you can’t take that back, you can’t go backwards and change it, so I definitely think it was the right thing to do and I guess I’m quite grateful for, I mean it wasn’t, it wasn’t pushed, but it was recommended (Sophie)*
	6.2 Regrets about decision
	*I wish I had seen him more (Kate)*

### Superordinate theme 1: Characteristics of contact

Whilst acknowledging that a stillbirth is a devastating experience for women, their partners and the extended family, the participants in this study described the experience of spending time with their baby as a process. Participants identified two main characteristics of contact with their stillborn baby: having time and shared experience.

A number of mothers emphasised the importance of having time with their baby:

*It was quite nice to have that time with her*, *looking back on it now. Even thinking about it at the time… Yes, it was so horrendous and so heart breaking, I’m glad we did it and spent time with her (Olivia)*

Although it was extremely difficult at the time, spending time with their baby was a cathartic experience and the majority of mothers valued the time they had. Many participants also spoke about being able to share their experience with their partner and also with parents and members of their extended family.

…Important everyone else got to see him because they are so close to me, and they were so close to me throughout the pregnancy as well. And they are excited about it. Yeah. Yeah I just wanted them to see how real he was. I wanted to make sure that anyone who wanted to hold him had held him (Victoria)

Involving those around them after the stillbirth was an important part of the process for mothers.

### Superordinate theme 2: Physicality

The theme physicality was a key theme that emerged from the transcripts. All participants expressed concerns about the physical appearance of their stillborn child prior to seeing their baby, and a number reported feeling relieved when they saw their baby looked normal and resembled other family members. However, for a few mothers, the appearance of their baby was quite disturbing.

Several participants described their fear and apprehension about seeing a dead body. They expressed concerns about what the baby would look like, as well as worries about how they would feel in response to being faced with a dead child.

I don’t know what I expected from touching her, I was so worried about touching a dead body, umm, and then to feel the cool skin was quite hard, it was quite waxy (Olivia)

Consequently, a number of participants felt a great sense of relief when they realised their baby was well-formed.

Just to see that he was just perfect, you know I checked everything out, his skin was lovely, he had ten fingers and ten toes (Julia)

Several participants talked about identifying family traits in their stillborn baby.

Her feet, they were like her dad’s, she had big toes (laughs) it was just the fact she was so perfectly formed, all the creases on her hands and feet, and the nails and the hair starting to come through and stuff like that (Katherine)

However, a few participants spoke about the damage and deterioration to their baby’s body and the negative implications that this had for them:

Unfortunately because she’d been inside me for some time and it was a pretty horrible forceps delivery in the end, had a bit of a problem in getting her out, a lot of the skin had come off so all down her side there was no skin and some of her arms and her face um and (partner) found that quite difficult. So when I was bathing her it was like ‘I don’t know how you can do that, I don’t know how you can do it’ (Chloe)

Whilst most mothers found the physical appearance and seeing their stillborn baby a positive experience, allaying fears of seeing the dead body and the excitement of comparing the baby to family members, some mothers whose baby’s body had been damaged or deteriorated struggled with seeing or holding their baby.

### Superordinate theme 3: Emotional experience

Every participant spoke at length about the emotional impact of having a stillbirth, including feelings of maternal pride at having a child, the grief they felt at losing their child and the positive process that followed for some mothers. Whilst acknowledging intense sadness and loss following a stillbirth, a number of mothers described how the experience of seeing their baby had been a positive one and the fond memories they held of the time they spent with their baby.

It was (reassuring), and it wasn’t what I expected at all and it was fine…nice in a way because we’ve got no other memories apart from me being pregnant and feeling her move inside me, we’ve got nothing else at all because she didn’t breathe, she didn’t have a life, so to have those memories is quite nice really (Olivia)

Some mothers also spoke of their pride at becoming a mother, and a positive change in how they felt about the stillbirth at the point of holding their baby for the first time.

Distraught, heartbroken, umm, angry, and then I held him and then it sort of all seemed to have gone away, and I started feeling happy. I managed to actually have a child which is more than most people get (Rebecca)

When looking back and describing the experience of spending time with their baby, the words “distraught”, “heart-breaking”, “angry” “pain” and “helpless” were over-riding expressions linked to grief used by participants. One mother reported:

Something I’ll never forget as long as I live. I miss her every day. I don’t think the pain will ever go. She’s somebody I wanted in my life so badly (Hannah)

Having a stillbirth was a highly emotional experience for mothers. All mothers talked about the intense pain and grief they felt at losing their child, but for some this was combined with a strong sense of maternal pride and the time spent with their stillborn baby was experienced as positive.

### Superordinate theme 4: Surreal experience

As well as finding the experience of seeing their baby after the stillbirth emotional, a number of participants also described the experience as surreal. Several mothers described feelings of disbelief:

I didn’t want to hold him, and I think that was almost upholding the illusion that he was alive in this basket, and if I held him it would be obvious that he wasn’t alive, and looking at him in the basket it was like he was asleep (Claire)

Other participants reported feelings of dissociation and described the experience as “*blurred like it was a dream (Victoria)*” and “*like looking at a doll.....not a baby. Not like mine but somebody else’s*” (*Sarah*). Similarly, some spoke about feeling disconnected from what they had experienced:

I guess the whole thing feels like it’s happened to somebody else. And you feel almost like it’s an out of body experience. And I guess the shock of it from the start is very much like this has happened to somebody else. I haven’t even been pregnant (Sophie)

For a few mothers, having a stillbirth resulted in strong feelings of disconnection from the world around them.

### Superordinate theme 5: Finality

Another key theme that emerged from the transcripts was the concept of finality. Participants talked about feeling sorry and needing to say goodbye to their child, as well as the realisation and acceptance that their child had died.

One participant explained “*I needed to say goodbye to her and I needed to say sorry*” (*Olivia*), sentiments echoed by a number of other mothers when looking back at the time they spent with their baby:

I got to say goodbye to him, that he was my baby, whether he was alive or dead. That everyone got to see him. Got to touch him (Victoria)

Participants also reported that seeing their baby following a stillbirth “*helps you to cope with what’s happened*” (*Rebecca*), as well as to accept that the baby had died:

It helped me to realise that she was dead. I think had we not seen her, err, it was a very, very real thing to have a dead body with you and yeah she’s dead, you know what else could she be, here she is, and if I hadn’t had seen her I'd be thinking ‘well is the doctor telling me the truth, is she dead, is somebody kidnapped her and bringing her up somewhere else’ you know that was all it as well. Umm, yeah I had forgotten that actually, I did think that at the time that it was quite important to see her (Olivia)

Having the opportunity to say goodbye and to see their baby brought about a sense of finality that for many mothers contributed to the healing process. It was something they felt they needed to do in order to move forward.

### Superordinate theme 6: Decision

When mothers looked back at how they felt about the decision to spend time with their stillborn baby, they either felt satisfied with the decision they had made to see their child or they had strong regrets about the decision they made at that time that were linked to the choice of not holding their baby. Most mothers reported feeling satisfied with the time they spent with and decision to see their baby.

I wouldn’t have done anything differently um I definitely would have seen her. And I guess I almost can’t believe I didn’t want to, it would have been quite hard not to have seen her. It definitely helped… I think I would have felt worse now if I hadn’t, you can’t take that back, you can’t go backwards and change it, so I definitely think it was the right thing to do and I guess I’m quite grateful for, I mean it wasn’t, it wasn’t pushy, but it was recommended (Sophie)

However, some mothers expressed regrets about the decision they took to not hold their stillborn baby:

I do I regret not holding him, and I think I regret not holding him purely because I never held him. Now, you know, I do regret not holding him. I think I should have been braver, but it’s very easy to say that in hindsight. Cause at the time couldn’t so. And maybe I was right at that time, cause if I had of held him I would have actually felt that physical sense of not having my baby in my arms. So perhaps it was a sort of self-preservation defence mechanism kicking in (Claire)

One mother also spoke of her sadness that her partner chose not to see their stillborn baby and the loss of shared experience as a family.

My partner – he didn’t have any contact with him, he didn’t want to hold him, umm just feel quite sad that he didn’t. He said that he would rather remember him in his head rather than the way he looked. I wasn’t going to pressurise him into doing anything he didn’t want to but now, looking back, I kind of wish we had a photo together or something (Kate)

For the majority of mothers, the decision to see their stillborn baby was crucial to their ability to accept what had happened and to say goodbye, and then to move forward.

## Discussion

This study explored in detail mothers’ qualitative experience of spending time with their stillborn baby to enhance understanding of the psychological and emotional impact of seeing and holding the baby following a stillbirth. Mothers talked about the importance of having time with their baby and being able to share the experience with those around them. A number of women expressed concerns about seeing a dead body and the physical appearance of their stillborn baby. While most parents felt relief that their baby was well-formed, identifying family traits and making memories, for some mothers the damage and deterioration to their baby’s body had significant negative implications. Mothers spoke at length about the emotional impact of having a stillbirth, grappling with feelings of maternal pride and grief. For a few mothers, the stillbirth resulted in feelings of disbelief and dissociation from the world around them. Having the opportunity to say goodbye and seeing the baby helped to bring about the realisation and acceptance for many mothers that their baby had died. Although the majority of mothers felt satisfied with their decision to see their baby following the stillbirth, a few mothers reported strong feelings of regret about the decision they took to not hold their stillborn baby.

Having contact with their stillborn baby provided mothers with time to process what had happened and to build memories, often sharing the experience with partners and other family members. A recent study emphasized the importance of sharing memories of the stillborn baby to aid psychological adjustment [41]. Mothers expressed feeling proud, and identifying family traits helped them to shift from feelings of desperation and heartbreak to happiness at the child they had produced. This is a new finding not previously reported. Seeing the baby also helped to bring about the realisation of the stillbirth, and helped mothers to accept that their baby had died. Furthermore, it provided mothers with the opportunity to say goodbye.

The majority of mothers felt satisfied with their decision to spend time with their stillborn baby. However, some mothers had deep regrets about some aspects of their contact with their baby, wishing that they had held them or for their partners to have spent time with them. The few mothers who had chosen not to hold their baby described regretting this decision three months later. This finding is in line with a recent study [27] and is in contrast with the recommendation that 'carers should avoid persuading parents to have contact with their stillborn baby, but should strongly support such desires when expressed' [42]. It might, in fact, be appropriate for staff to make a strong case to parents for seeing and holding their stillborn baby. Consistent with previous research [18-20,27], most of the mothers in this study found spending time with their stillborn baby helpful.

The results highlight a range of issues particularly relevant to health professionals working with mothers following stillbirth. Several mothers talked about the fear they felt about what the baby would look like, as well as fear of seeing a dead body in general. It is possible that this might prevent some from choosing to see their baby. Therefore, these fears should be sensitively explored and ways of coping should be discussed. Although many mothers felt relieved when they saw the baby was well formed, for others it was quite upsetting to see the damage and deterioration to their stillborn baby. It may therefore be important for health professionals to sensitively prepare mothers and fathers in cases where the body of their stillborn baby is damaged; an aspect that has not been highlighted in previous research. A number of mothers talked about feelings of helplessness, intense sadness and pain which were exacerbated when seeing their stillborn baby. Particularly in those cases, professional support and opportunities for expressing these emotions need to be provided.

For a few mothers, the experience of seeing their baby was associated with feelings of disbelief and in some cases dissociation, with mothers unable to come to terms with what had happened. The role of dissociation during the contact with the stillborn baby has not previously been highlighted and requires further investigation. It is possible that certain aspects of how the contact with the baby is facilitated (e.g. how parents are prepared by staff, length of time spent with the baby, how the baby is presented by staff etc.) might increase the risk of dissociation occurring. Mothers and fathers who have dissociative experiences during contact with their stillborn baby might be more vulnerable to developing subsequent symptoms of posttraumatic stress disorder or traumatic grief [43]. It is therefore recommended that these couples are followed up and screened for symptoms of posttraumatic stress approximately one month after stillbirth.

Clearly, it is important to involve parents in all decisions regarding their contact with their stillborn baby. Giving parents choices by engaging in discussions about the possible benefits and risks (particularly in those cases where the body is damaged) of seeing their stillborn baby is crucial. What was reported to be helpful to some mothers who experienced stillbirth in this study was not always helpful to others. This is a view echoed by Reynolds [44], who emphasised that “everyone grieves differently in their own way and in their own time… we cannot offer a one size fits all approach to psychosocial intervention following stillbirth” (page 87). Nevertheless, our data seems to suggest the notion that it may be appropriate for staff to make a strong case to parents for having contact with their stillborn baby. Furthermore, recent evidence [45] suggests that “mothers feel more natural, good, and less frightened and uncomfortable when they see and hold their stillborn baby if providers assumptively offer the baby, rather than asking” (page 248).

Interpretations of the results are limited by the fact that all women in the sample had chosen to see and the majority had chosen to hold their stillborn baby. It is therefore not possible to draw conclusions about the experience of women who had not chosen to see their baby. The majority of mothers in this study were married and few mothers had a household income of under £30,000. Therefore, the transferability of our findings to low income families and unmarried mothers may be limited. Furthermore, mothers reported retrospectively about their contact with their stillborn baby three months after it had taken place, so there is the potential that some memories may have been altered or distorted with time. A strength of the study is that all participants responded well to the individual interviews that were conducted in their own environment [46]. Conducting interviews in women's homes meant that women could be more relaxed in their own environment and were therefore more willing to talk freely [47]. Furthermore, all authors were involved in the interpretive process, thereby enhancing the authenticity of the accounts generated.

## Conclusions

The majority of mothers in this study described the experience of their contact with their stillborn infant as positive and important and felt that they had taken the right decision in doing so. However, some issues were identified that warrant the attention of health professionals working with this population, such as mothers' fear of seeing a dead body or seeing the damaged or deteriorated body of their stillborn baby. For some mothers, intense feelings of helplessness, sadness and pain were exacerbated when seeing their stillborn baby and some reported dissociating when seeing their baby. In these cases, preparation before the contact with the baby, professional support during the contact, and professional follow-up are crucial in order to prevent the development of mental health problems. Nevertheless, even in cases where mothers experienced intense distress during the contact with their stillborn baby, they still described that having had this contact was important and that in hindsight they felt they had taken the right decision. These findings indicate a need for giving parents an informed choice by engaging in discussions about the possible benefits and risks of seeing their stillborn baby, rather than rigidly following perinatal bereavement protocols and guidelines.

## Competing interests

The authors declare that they have no competing interests.

## Authors’ contributions

KR conducted the interviews and data analysis and drafted the manuscript. CRC participated in the data analysis and drafting of the manuscript. KMc participated in the conception, design and coordination of the study, data collection, data analysis and commented on the manuscript. AH conceived of the study and its design, coordinated the data collection, participated in the data analysis and co-wrote the manuscript. All authors read and approved the final manuscript.

## Pre-publication history

The pre-publication history for this paper can be accessed here:

http://www.biomedcentral.com/1471-2393/14/203/prepub
